# Soil types create different rhizosphere ecosystems and profoundly affect the growth characteristics of ratoon sugarcane

**DOI:** 10.3389/fmicb.2025.1541329

**Published:** 2025-07-09

**Authors:** Xiaoming Wang, Junjun Ma, Chaoxian Fang, Jinghuan Zhu, Shilong Wang, Zuli Yang

**Affiliations:** ^1^Key Laboratory for the Green and Efficient Production Technology of Sugarcane, Guangxi Science & Technology Normal University, Laibin, China; ^2^Institute of Analysis and Testing, Beijing Academy of Science and Technology (Beijing Center for Physical & Chemical Analysis), Beijing, China; ^3^Laibin Comprehensive Experiment Station of National Sugar Industry Technical System, Laibin Academy of Agricultural Sciences, Laibin, China

**Keywords:** ratoon sugarcane, rhizosphere soil, rhizosphere microbes, soil types, growth characteristics

## Abstract

**Introduction:**

Rhizosphere ecological factors play a crucial role in the soil feedback function of ratoon sugarcane. However, limited information exists regarding the differences and relationships among these factors across various soil types (sandy, loam, and clay) and their impact on sugarcane growth and yield characteristics. This study aims to address this knowledge gap by investigating the rhizosphere ecological dynamics of ratoon sugarcane in different soil types.

**Methods:**

A combination of biochemical experiments and high-throughput sequencing was employed to analyze the rhizosphere ecological factors of ratoon sugarcane. The study examined biochemical function- and enzymatic activity-related factors in the rhizosphere soil, as well as the microbial community composition and their relationships with sugarcane growth and yield characteristics.

**Results:**

Biochemical function- and enzymatic activity-related factors in the rhizosphere soil were generally positively correlated with each other and with sugarcane growth characteristics. For instance, soil respiration and soil catalase activity showed significant positive correlations with theoretical sugarcane yield (correlation coefficients of 0.773, *p* < 0.05 and 0.863, *p* < 0.01, respectively). Symbiotic relationships were observed between the rhizosphere soil microbial and root system endophyte communities. Functional differences in microbial communities among different soil types were significant (*p* < 0.05), indicating that soil type strongly influences the functional expression of rhizosphere soil microbial communities. The abundance of bacteria and fungi in the ratoon sugarcane rhizosphere soil was negatively and positively correlated with most soil biochemical functions, respectively. Additionally, correlations existed between the abundance of endophytic bacteria and fungi in the root system and sugarcane yield. Eleven soil biochemical and functional factors were positively correlated with theoretical sugarcane yield and theoretical sugar yield indicators.

**Discussion:**

Our findings suggest that different feedback directions and correlation strengths exist between sugarcane growth characteristics and various ecological factors in their rhizosphere across different soil types and spatial scales. These results provide insights into the complex interactions between sugarcane and its rhizosphere environment, highlighting the importance of soil type in shaping these interactions. The study offers guidance for improving soil microbial community structure to enhance sugarcane growth and yield, serving as a valuable reference for soil management in sugarcane fields.

## 1 Introduction

Correlations exist between soil ecological factors and the growth characteristics of crops. In sugarcane, *Saccharum officinarum*, many rhizosphere ecological factors correlate with the growth characteristics of the plant, including allelochemicals, physicochemical properties, the heavy metal content and biochemical functional factors of the soil, rhizosphere soil microbes and root system endophytic microbes (Wang et al., 2024). These growth characteristics are agronomic indicators related to sugarcane yield and quality formation during the maturation stage of the plant, which include the number of green leaves, plant height, stem diameter, effective stem count, single stem weight, theoretical sugarcane yield, sucrose content and theoretical sugar yield (Xiao et al., 2023). Sugarcane growth and development have specific demands on the nutrient elements within the soil. With the long-term adoption of no-till practices in the cultivation of ratoon sugarcane, rhizosphere ecological factors may evolve in specific directions over time. These include deficiencies in essential nutrient elements and the accumulation of allelochemicals secreted or decomposed by the root system, which may cause the deterioration of overall rhizosphere ecological factors, thereby affecting the normal growth and development of subsequent sugarcane crops. For instance, the stress resistance of sugarcane may be reduced during the growth process, ultimately impacting the formation of agronomic traits, yield, and quality (Valentina et al., 2021; Yang et al., 2021; Zhang et al., 2021). It has been shown that the application of goat manure and sugarcane straw in soil can affect the colony structure in soil, change the soil pH value and significantly improve the soil nutrients, especially C, N and P (Tayyab et al., 2018).

Soil bacteria and fungi are closely related to soil properties. A large number of bacteria and fungi associated with the quality of sugarcane have been found in the process of continuous sugarcane planting, which is of great significance for studying obstacle mechanisms in continuous sugarcane planting (Pang et al., 2021). In China, sugarcane is mainly planted in Guangxi, Yunnan, Guangdong and Hainan, and these regions have different soil types, including sandy soil, loam soil and clay soil (Luo et al., 2015). A study by Fallah et al. (2023) explored bacteria biocontrol agents, biochar and secondary metabolites to rejuvenate ratoon sugarcane traits and stimulate soil fertility, and this demonstrated how the multifunctionality of plant secondary metabolites (BCAs-BC) interactions remediated soil fertility and suppressed the presence of pathogens/parasites (such as *Exserohilum, Aphis gossypii* Glover, and *Colletotrichum*), thereby rejuvenating ratoon sugarcane growth and productivity. Smith-Ramesh and Reynolds (2017) argued that three broad factors drive plant–soil feedback, namely soil microbes (including pathogens and symbiotic microbes), soil physicochemical/biochemical factors and the secondary metabolites of organisms. In addition, a study by Liu et al. (2017) found that long-term continuous cropping of potatoes led to a decrease in single tuber weight and a reduction in the tuber yield by up to 30%. Continuous potato cropping causes a significant decrease in the sink capacity and source activity of plants, which shortens the dry matter rapid growth period, reduces and significantly increases the rate of dry matter accumulation and the proportion allocated to the root organs, respectively, and ultimately results in lower yields. During tuber formation, dry matter is stored in the tubers by the nutrient organs transport before flowering, and the direct input of assimilates to the tubers is decreased after flowering. Kuerban et al. (2020) found that continuous cropping caused growth inhibition, yield reduction and growth cycle delays in sweet sorghum; this was manifested as significant decreases in plant height, stem diameter, single plant weight and single stalk weight, and a delay in the maturation period after 2 years of continuous cropping.

Yang et al. (2019) found that sugarcane plants infected with ratoon stunting disease exhibited a decrease in bacterial diversity and richness in the rhizosphere soil compared with those in the uninfected plants. The dominant bacterial community structure also changed considerably at the phylum and genus taxonomic levels. With long-term continuous cropping, allelopathic and autotoxic substances can alter the ecological environment of the rhizosphere (Wang et al., 2022). For instance, these substances can change the pH, the ability to provide nutrient elements, the microbial community structure and enzymatic activities of the soil and the resistance of plants to diseases and pests; this consequently affects the physiological development, yield, and quality of the crops (Liu et al., 2021; Chen et al., 2022).

The plant–soil feedback loop is driven by the intricate interplay of complex factors including rhizosphere physicochemical properties, soil biochemical functions, soil biological community structures, endophytic community compositions and their secondary metabolites (Chen et al., 2021; Wang et al., 2021b). The diversity of soil types (sandy, loam and clay) in the Laibin sugarcane growing area of Guangxi, China reflects its complex geomorphology and ecological conditions. Different soil types influence rhizosphere environment, nutrient utilization and root development, leading to differences in sugarcane yield and planting strategies. As it is of great significance to study the growth of sugarcane on different soil types, we analysed the rhizosphere ecological characteristics of ratoon sugarcane (same variety) grown on different soil types (across spatial scales) under identical climatic and environmental conditions. We investigated the correlations among ecological factors (such as rhizosphere soil biochemical, functional factors and soil microbes) and the diversity, community structure and function of root system endophytes across spatial scales (different soil types). Additionally, we analysed the relationships between these ecological factors and sugarcane growth characteristics. Our results serve as a reference for improving soil conditions in sugarcane fields.

## 2 Materials and methods

### 2.1 Overview of sampling area

The sampling area was within the National Modern Agricultural Industrial Park in Laibin City, Guangxi Province, China (N 23°48′, E109°12′), located in central Guangxi downstream of the Hongshui River. Laibin City is the second largest sugarcane planting city in China, with a planting area expected to reach 130,000 hectares by 2024. The high-quality sugarcane varieties and circular economy model of Laibin National Modern Agricultural Industrial Park provide reference for other regions. The research site is located in the transitional zone between the southern and central subtropical climatic zones. The average annual temperature is approximately 21°C, with relatively dry winters and springs and abundant rainfall during summer and autumn. The average annual precipitation is approximately 1,300 mm, with an average of 1,500 h of sunshine annually. The research site comprises an area of 10 hectares and is characterised by relatively flat terrain. Three different soil types were selected in the experimental area: sandy soil (mainly sand (0.05–2 mm), accounting for about 50%), loam soil (mainly coarse soil powder (0.1–0.5 mm), accounting for about 40%, and fine sand less than 30%), and clay soil (mainly fine powder (<0.05 mm), accounting for more than 30%). The soil types in the sugarcane planting area of Laibin City are mainly sandy soil, loam soil and clay soil, presenting a complex and diverse terrain, landforms and soil ecological conditions. Different soil types create different rhizosphere ecological environments and nutrient supply conditions, leading to differences in sugarcane yield and planting strategies. The preceding crop in each plot was sugarcane. New sugarcane (Guiliu 05136 variety) was planted in the three different soil types in February 2019. The field management measures of sugarcane were the same. Sugarcane was harvested manually in January 2020. After harvest, the perennial roots were left, representing the first-year perennial sugarcane roots. The sugarcane field was cleaned immediately and managed according to the unified perennial root sugarcane cultivation measures.

Five sample plots, each measuring 10 m × 10 m, were established in the plots containing each soil type, totalling 15 sample plots. Sample collection was conducted in the first-year sugarcane ratoon plot containing sandy, loam or clay soil. Field investigations were conducted during the sugarcane harvest period (mid-November 2020) to obtain samples and data from ratoon sugarcane rhizosphere sandy (SS), loam (LS), and clay (CS) soils, and ratoon sugarcane root systems in sandy (GSS), loam (GLS), and clay (GCS) soils, as well as the sugarcane growth characteristics. The fertilization regime involved two applications (Wang et al., 2019): a base fertilizer of compound fertilizer (N:P:K = 15:15:15; total nutrients ≥45%) at 375 kg/hm^2^ and urea at 225 kg/hm^2^, followed by a mid-tillage application (early July annually) of compound fertilizer (375 kg/hm^2^), urea (525 kg/hm^2^) and potassium chloride (225 kg/hm^2^). The weather and time during sample collection were consistent; samples were collected in the morning when the weather was fine and the wind scale was less than or equal to level 3.

### 2.2 Sample collection procedure

Soil sample collection (Panke-Buisse et al., 2015): five sample plots were set for each soil type (five replicates). Refer to the “Technical Specification for Soil Testing and Formula Fertilization (2011 Revised Edition)” issued by the Ministry of Agriculture of China. Five soil samples were collected from each sample plot using the “S”-shaped five-point mixed sampling method, and the mixed samples formed a replicate. After removing surface weeds and scraping off the topsoil, rhizosphere soil was collected from 10 to 20 cm below the ground. Five soil samples were collected from each sample plot using the “S”-shaped five-point mixed sampling method, and the mixed samples formed a replicate. After excavating the sugarcane roots, they were gently shaken to remove large clumps of soil, loose soil, and debris. A sterile brush was then used to collect residual soil from the root surface. Soil samples from multiple points within the plot were combined in equal volumes, mixed thoroughly and placed in sterile bags before being transported to the laboratory on dry ice for further processing. A portion of the samples was stored in a refrigerator at 4°C, and soil biochemical and enzyme activity tests were completed within 1 week. Another portion was rapidly frozen with liquid nitrogen and stored in a freezer at −80°C to determine the microbial diversity of rhizosphere soil (16S rRNA/ITS).

When collecting sugarcane rhizosphere soil samples, sugarcane root samples were also collected within the plots using the “S-shaped” five-point composite sampling method. We randomly selected 3–5 healthy root tissues of varying lengths as one sample (approximately the length of fingers) and ensured that the soil adhering to the root surface was removed. The samples were placed in sterile bags and transported to the laboratory on dry ice. The root samples were first rinsed with sterile water for 30 s, followed by immersion in 70% ethanol for 2 min. Finally, the roots were washed thrice with sterile water for surface sterilization (Edwards et al., 2015). The pretreated root samples were then placed in sterile EP centrifuge tubes (with screw caps), quickly frozen with liquid nitrogen, and stored in a − 80°C refrigerator for root microbial diversity (16S/ITS) testing.

### 2.3 Analytical parameters and methods

To determine the biochemical functional activities in the sugarcane rhizosphere soil, we measured ammonification, nitrification and nitrogen fixation activities following the methods described by Lin (2010) and Xu and Zheng (1985). Soil respiration was determined using a soil carbon flux measurement system to assess the respiratory activity.

Various analytical approaches were used to determine enzyme activities in the sugarcane rhizosphere soil. The activity of polyphenol oxidase (PPO) was measured using the pyrogallol colourimetric method (enzyme assay kit, produced by Shanghai Youxuan Biotechnology Co., Ltd., China, and refering to the instructions of Sinobestbio enzyme activity detection kit, using the microcalorimetry method, same as below), where the production of 1 mg of pyrogallol per gram of sample per day was defined as one unit (U) of enzyme activity. The activity of CAT, peroxidase (POD), ACP, cellulase (CL), sucrase (SC), UE and fluorescein diacetate hydrolase (FDA) were determined using the microquartz colourimetric (enzyme assay kit), microglass colourimetric (purpurogallin) (enzyme assay kit), disodium phenyl phosphate (enzyme assay kit), 3-dinitrosalicylic acid colourimetric (enzyme assay kit), 3,5-dinitrosalicylic acid colourimetric (enzyme assay kit), indophenol blue colourimetric (enzyme assay kit) and microquartz colourimetric dish (enzyme assay kit) methods, respectively, where the production of 1 μmol of H_2_O_2_, 1 mg of purpurogallin, 1 nmol of phenol, 1 mg of glucose, 1 mg of reducing sugar, 1 μg of NH_3_-N and 1 μmol of fluorescein per gram of sample per day was defined as one unit (U) of enzyme activity, respectively.

Data on agronomic traits, the yield and sugar content of sugarcane were collected in corresponding sample plots according to different treatments at the designated sampling sites. During harvesting, the data collection method involved randomly selecting 10 sugarcane plants from each replicate plot to conduct a sucrose content analysis, which was conducted using the double polarisation method (Pu and Lin, 2015). Additionally, for each replicate plot, three rows of sugarcane were randomly selected (with each row measuring 3 m in length) to estimate the field yield of sugarcane. The collected data included the number of green leaves, plant height (measured 30 cm below the thickest part of the sugarcane stalk), stem thickness and effective stem count. The average collected data values of each group were used for further analysis. The theoretical sugarcane (t/hm^2^) and sugar (t/hm^2^) yields were calculated as follows: single stalk weight (kg) = (stem thickness (cm))^2^ × plant height (cm) × 0.7854/1,000; theoretical sugarcane yield (t/hm^2^) = single stalk weight (kg) × effective stem count (stems/hm^2^)/1,000; theoretical sugar yield (t/hm^2^) = sucrose content (%) × theoretical sugarcane yield (t/hm^2^).

### 2.4 Microbial DNA extraction and sequencing

The extracted DNA from the root-zone soil and root samples was stored at −80°C and then subjected to DNA extraction, PCR amplification and Illumina MiSeq sequencing. DNA extraction and PCR amplification were performed according to the instructions from the E.Z.N.A.^®^ soil DNA kit (Omega Bio-tek, Norcross, GA, United States). The quality of DNA extraction was verified using 1% agarose gel electrophoresis, and the DNA concentration and purity were measured using a NanoDrop 2000 spectrophotometer. For the 16S rRNA gene region, and for the ITS1–ITS2 region, the 338F (5′-ACTCCTACGGGAGGCAGCAG-3′) and 806R (5′-GGACTACHVGGGTWTCTAAT-3′), and ITS1F (5′-CTTGGTCATTTAGAGGAAGTAA-3′) and ITS2R (5′-GCTGCGTTCTTCATCGATGC-3′) primer pairs were used for PCR amplification, respectively. The PCR products from the same sample were pooled and recovered using a 2% agarose gel, purified using the AxyPrep DNA Gel Extraction Kit (Axygen Biosciences, Union City, CA, United States) and checked for quality using 2% agarose gel electrophoresis. The recovered products were quantified using a Quantus™ Fluorometer (Promega, Madison, WI, United States). Library preparation was performed using the NEXTFLEX^®^ Rapid DNA-Seq Kit, and sequencing was conducted using the Illumina MiSeq PE300 platform. The sequencing work was conducted by Shanghai Majorbio Bio-pharm Technology Co., Ltd., Shanghai, China (Dineen et al., 2010; Zhang et al., 2016). The sequencing reads of those samples were submitted to NCBI- Sequence Read Archive (SRA) and obtained the accession code PRJNA1247173^1^ and PRJNA1249922.^2^ Q20 98.02% percentage above were obtained after removing adapters, low-quality regions (<100 bp) and possible contamination from 5 biological replicates of the 30 soil and root system samples.

### 2.5 Data processing methods

#### 2.5.1 Non-microbial data analysis method

Data statistics and analysis results were recorded using WPS Office 2016 XLSX spreadsheets. Minitab 16 was used to perform the analysis of variance (ANOVA) (including standard deviation and *p*-value analysis for significance of differences). Experimental data are presented as the “mean ± standard deviation.”

#### 2.5.2 High-throughput sequencing data analysis method

The fastp software was used to perform quality control on paired-end raw sequencing sequences and the FLASH software for merging in accordance with the following procedure: (1) Bases with a read tail quality value below 20 were filtered, and a 50-bp window was thus set. If the average quality value within the window was lower than 20, the back-end bases were truncated from the window, and the reads below 50 bp after quality control were filtered and those containing N bases were removed. (2) According to the overlap relationship between the PE reads, the paired reads were spliced (merged) into one sequence. The minimum overlap length was 10 bp. (3) The maximum mismatch ratio allowed in the overlap region of the spliced sequence was 0.2, and the unmatched sequences were screened out. (4) The samples were distinguished according to the barcode and primers at the beginning and end of the sequence, and the sequence direction was adjusted. The number of mismatches allowed by the barcode was 0, and the maximum number of primer mismatches was 2. The UPARSE software was used to perform operational taxonomic unit (OTU) clustering on the quality control-spliced sequences based on 97% similarity and eliminate chimeras. The RDP classifier was used to compare the Silva 16S rRNA gene and the UNITE 8.0 ITS databases for taxonomic annotation of OTU species, with a confidence threshold of 70%. Microbial community is based on the R package “Phyloseq” for sample homogenisation of total abundance. Alpha and beta diversity analyses of microbial communities, correlation analyses between environmental factors and microbial communities, and Spearman correlation analyses were performed using the R language (version 4.2.1) and the “vegan” package. Dissimilarities among the microbial community structures of the different groups were tested and analysed using three methods: permutational multivariate analysis of variance (PERMANOVA or ADONIS), analysis of similarities (ANOSIM), and multiple response permutation procedure (MRPP). The significance of community composition disparities and Generalised Additive Model (GAM) were analysed using the R package “vegan” and “mgcv,” respectively. Then, the Mantel Test was conducted using the mantel function from the R package “vegan.” Species composition stacked plots were generated using the Python library “Pandas” for “Groupby” analysis, and GraphPad Prism 8 was used for plotting and significance calculations (based on Student’s *t*-test and ANOVA). Functions of all microbial communities were predicted using Tax4Fun2.

## 3 Results

### 3.1 Soil biochemical function in the sugarcane rhizosphere on different soil types

#### 3.1.1 Biochemical activities of sugarcane rhizosphere on different soil types

The soil respiration of LS was significantly higher than that of SS and CS (*p <* 0.05), and the difference in soil respiration between SS and CS was not statistically significant (Table 1). LS and SS showed significantly higher ammonification and nitrogen fixation rates than those in SS and CS (*p* < 0.05) and CS (*p* < 0.05), respectively. Ammonification and nitrogen fixation in LS were 152.67 and 248.26% higher than those in CS, respectively. SS and LS showed significantly higher nitrification rates than those in CS (*p* < 0.05), although the difference in nitrification between SS and LS was not statistically significant. Nitrification in SS and LS was 158.13 and 144.54% higher than those in CS, respectively. These results indicate that the respiratory, ammonification, nitrification and nitrogen fixation activities of LS were high, the nitrification activity of SS was high and the biochemical activities of CS were comparatively weaker. These contrasting results may be related to differences in the physicochemical properties or microbial diversity among the different soil types.

**Table 1:** Biochemical activities of sugarcane rhizosphere within different soil types.

Soil type	Soil respiration	Ammonification	Nitrification	Nitrogen fixation
	μmol/(m^2^·s)	mg/kg	mg/kg	mg/kg
Sandy soil (SS)	1.68 ± 0.12 b	265.82 ± 9.66 b	940.1 ± 63.0 a	70.97 ± 3.66 b
Loam soil (LS)	1.96 ± 0.11 a	347.41 ± 13.11 a	859.3 ± 21.2 a	88.01 ± 4.14 a
Clay soil (CS)	1.50 ± 0.11 b	227.56 ± 11.51 c	594.5 ± 33.3 b	35.45 ± 2.10 c

The data represent the mean ± standard deviation. Different lowercase letters after each factor in the same row indicate significant differences between different soil types at a significance level of 0.05.

#### 3.1.2 Enzymatic activities of sugarcane rhizospheres on different soil types

In this study, the differences in PPO and CL activities among the various soil types were not significant, with the activities of SS and LS being generally higher than those of CS (Table 2). The CAT and POD activities of LS were significantly higher than those of SS and CS (*p* < 0.05), and the CAT and POD activities of LS were 135.54 and 162.91% and 134.65 and 167.83% higher than those of SS and CS, respectively. The ACP and UE activities of LS were significantly higher than those of SS and CS (*p* < 0.05) and those of SS were significantly higher than those of CS (*p* < 0.05). The CAT and POD activities of LSs were 279.51 and 169.23% higher than those of CS. The invertase and hydrolase activities of LS were significantly higher than those of CS (*p* < 0.05). The enzymatic activities of LS were higher than those of SS, and those of SS were higher than those of CS, although the differences were not significant. Generally, the sugarcane rhizosphere soil enzymatic activities of different soil types exhibited a trend of LS > SS > CS, which may be related to differences in the physicochemical properties or microbial diversity among the different soil types.

**Table 2:** Enzymatic activities of sugarcane rhizosphere within different soil types.

Soil type	Polyphenol oxidase activity	Catalase activity	Peroxidase activity	Acid phosphatase activity	Cellulase activity	Invertase activity	Urease activity	Hydrolytic enzyme activity
	U/g	U/g	U/g	U/g	U/g	U/g	U/g	U/g
Sandy soil	25.47 ± 1.61 a	40.18 ± 4.57 b	28.83 ± 4.12 b	2454.0 ± 412.2 b	123.20 ± 19.64 a	203.58 ± 20.67 ab	82.22 ± 10.38 b	40.81 ± 5.06 ab
Loam soil	25.32 ± 2.28 a	54.46 ± 4.75 a	38.82 ± 5.55 a	3660.7 ± 627.7 a	132.38 ± 20.37 a	229.69 ± 21.36 a	102.47 ± 10.89 a	45.23 ± 5.96 a
Clay soil	22.12 ± 1.93 a	33.43 ± 5.54 b	23.13 ± 3.80 b	1309.7 ± 308.8 c	112.32 ± 18.64 a	174.01 ± 17.08 b	60.55 ± 7.63 c	33.76 ± 5.12 b

The data represent the mean ± standard deviation. Different lowercase letters after each factor in the same row indicate significant differences between different soil types at a significance level of 0.05.

#### 3.1.3 Agronomic traits, yield, and quality indicators of sugarcane grown on different soil types

The number of green leaves of sugarcane grown on LS was significantly higher (*p* < 0.05) than that on SS and CS (Table 3). The number of green leaves was higher in sugarcane grown on SS than that on CS, but not significantly. During the harvest period, the number of leaves of sugarcane grown on LS was significantly higher, which may be related to the stronger ability of the physicochemical factors in LS for sustained nutrient provision. The plant heights of sugarcane grown on SS and LS were not significantly different, although both were significantly greater than that on CS (*p* < 0.05). Furthermore, the mean plant height of sugarcane grown on SS and LS was approximately 17 cm greater than that grown on CS. Stem diameter, effective stem count, single stem weight and sucrose content did not differ significantly among sugarcane grown on different soil types, and the overall trend was LS > SS > CS. However, the sucrose content decreased in the order of SS > LS > CS; this may be attributed to the weaker water retention and supply abilities of SS, leading to the concentration and elevation of sucrose content during the harvest period due to the transpiration of water content from sugarcane. The theoretical yield of sugarcane did not differ significantly between sugarcane grown on SS and LS, although it was significantly higher in sugarcane grown on LS compared with that on CS (*p* < 0.05). The same trend was observed with respect to the theoretical yield of sugar: the theoretical sugarcane and sugar yields were, respectively, higher in sugarcane grown on SS (12.44 t/hm^2^ and 2.33 t/hm^2^) and LS (15.38 and 2.63 t/hm^2^) than that on CS (83.51 and 11.67 t/hm^2^). These results indicate that different soil types influenced sugarcane growth characteristics and sugar yield.

**Table 3:** Agronomic traits, yield and quality indicators of sugarcane grown in different soil types.

Soil type	Number of sugarcane leaves	Sugarcane plant height	Sugarcane stem diameter	Number of sugarcane effective stems	Single stem weight of sugarcane	Theoretical sugarcane yield	Sugar content of sugarcane	Theoretical sugar yield
	count/plant	cm	cm	plants/hm^2^	kg/plant	t/hm^2^	%	t/hm^2^
Sandy soil	6.80 ± 0.84 b	269.00 ± 6.00 a	2.69 ± 0.14 a	62,629 ± 1,032 a	1.53 ± 0.13 a	95.95 ± 6.47 ab	14.63 ± 0.45 a	14.06 ± 1.39 a
Loam soil	8.20 ± 0.84 a	270.67 ± 5.03 a	2.72 ± 0.14 a	62,779 ± 1,058 a	1.58 ± 0.14 a	98.89 ± 6.81 a	14.45 ± 0.41 a	14.30 ± 1.20 a
Clay soil	6.00 ± 0.71 b	253.67 ± 6.03 b	2.61 ± 0.11 a	61,365 ± 1,027 a	1.36 ± 0.08 a	83.51 ± 6.31 b	13.98 ± 0.35 a	11.67 ± 0.79 b

The data represent the mean ± standard deviation. Different lowercase letters after each factor in the same row indicate significant differences between different soil types at a significance level of 0.05.

### 3.2 Correlation analysis between non-microbial ecological factors and sugarcane growth characteristics

**Figure 1:**
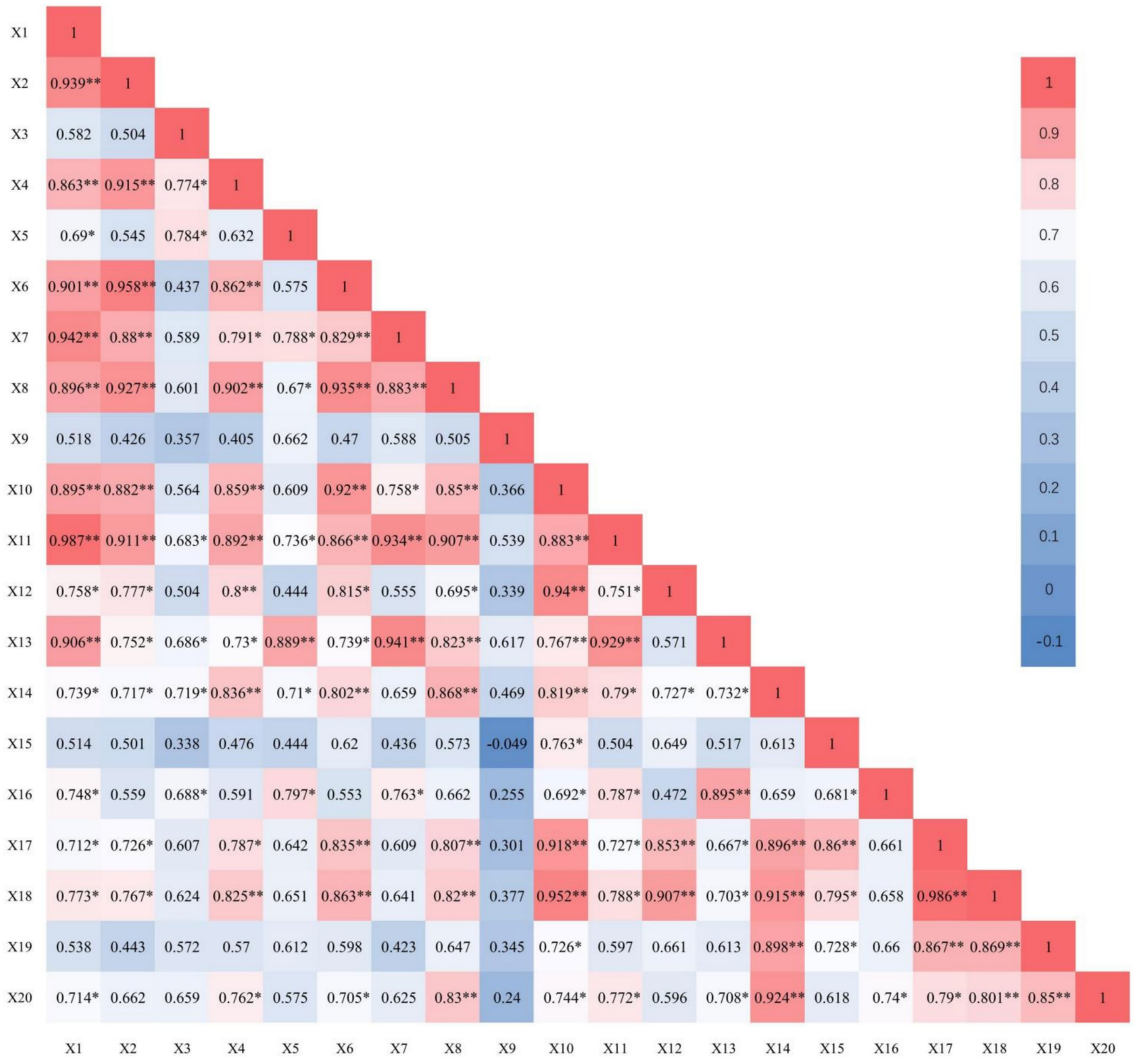
Correlation analysis between non-microbial ecological factors in rhizosphere soil and sugarcane growth characteristics. X1: Soil respiration; X2: Ammonification; X3: Nitrification; X4: Nitrogen fixation; X5: Polyphenol oxidase activity; X6: Catalase activity; X7: Peroxidase activity; X8: Acid phosphatase activity; X9: Cellulase activity; X10: Sucrose activity; X11: Urease activity; X12: Hydrolytic activity; X13: Number of sugarcane leaves; X14: Sugarcane plant height; X15: Sugarcane stem diameter; X16: Number of sugarcane effective stems; X17: Single stem weight of sugarcane; X18: Theoretical sugarcane yield; X19: Sugar content of sugarcane; X20: Theoretical sugar yield. * Significant correlation at *p* < 0.05, ** significant correlation at *p* < 0.01.

Figure 1 shows the correlation coefficients obtained by analysing various non-microbial ecological factors in sugarcane rhizosphere soil. The sugarcane growth characteristics measured in the current soil were correlated with each other and with the sugarcane growth characteristics.

Soil respiration, ammonification, nitrification and nitrogen fixation were positively correlated with soil enzymatic activities and sugarcane growth characteristics. For instance, soil respiration was highly significantly positively correlated with ammonification, nitrogen fixation, CAT activity, POD activity, UE activity of the soil and the number of green leaves of sugarcane (*p* < 0.01). Soil respiration was also significantly positively correlated with PPO activity, hydrolase activity of the soil and the effective stem count, single stem weight, the theoretical sugarcane yield and the theoretical sugar yield of sugarcane (*p <* 0.05). Soil ammonification was highly significantly positively correlated with nitrogen fixation, CAT activity, ACP activity and UE activity of soil (*p* < 0.01) and significantly positively correlated with the number of green leaves, plant height, single stem weight and the theoretical sugarcane yield of sugarcane (*p* < 0.05). Soil nitrification was significantly positively correlated with nitrogen fixation, PPO activity, UE activity of soil, plant height and the effective stem count of sugarcane (*p* < 0.05). Soil nitrogen fixation was highly significantly positively correlated with CAT activity, ACP activity, UE activity of the soil and the theoretical yield of sugarcane (*p* < 0.01), and significantly positively correlated with the POD activity of the soil, the number of green leaves, single stem weight and the theoretical sugar yield of sugarcane (*p* < 0.05).

Various enzymatic activities in soil exhibited positive correlations with each other and the sugarcane growth characteristics. Specifically, soil CAT, ACP, invertase, UE and hydrolase activities were strongly positively correlated with sugarcane growth characteristics, those of soil PPO and POD activities were weakly positively correlated, and soil CL activity was weakly positively correlated with all other enzymes and sugarcane growth characteristics.

Sugarcane growth characteristics showed strong positive correlations with each other (Figure 1). For instance, sugarcane plant height was highly significantly positively correlated with single stem weight, theoretical sugarcane yield, sucrose content and theoretical sugar yield (*p* < 0.01); sugarcane stem diameter was highly significantly positively correlated with single stem weight (*p* < 0.01); effective stem count was significantly positively correlated with theoretical sugar yield (*p* < 0.05); single stem weight was highly significantly positively correlated with theoretical sugarcane yield and sucrose content (*p* < 0.01); theoretical sugarcane yield was highly significantly positively correlated with sucrose content and theoretical sugar yield (*p* < 0.01); and the sucrose content of sugarcane was highly significantly positively correlated with theoretical sugar yield (*p* < 0.01).

### 3.3 Correlation analysis between microbial ecological factors and sugarcane growth characteristics

#### 3.3.1 Diversity and relative abundance of microbes

**Figure 2:**
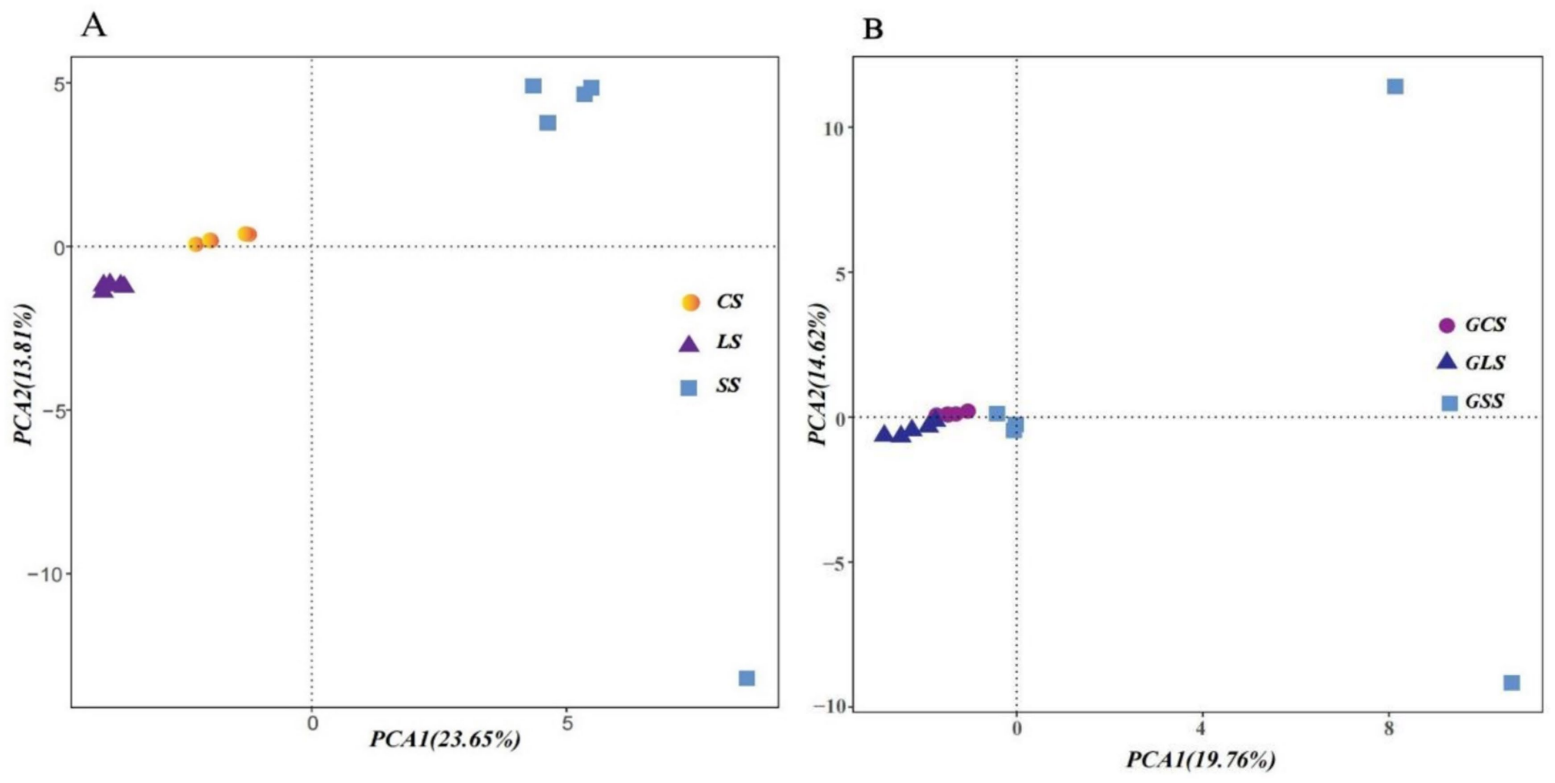
Principal component analysis (PCA) of rhizosphere soil microbial **(A)** and root system endophyte communities **(B)**. PC1 and PC2 represented the first and second principal component, respectively, whereas percentages indicated the explanatory power of these principal components on the dataset. The samples and data from ratoon sugarcane rhizosphere sandy (SS), loam (LS), and clay (CS) soils, and ratoon sugarcane root systems in sandy (GSS), loam (GLS), and clay (GCS) soils. Each point in the figure represented a biological sample, with samples belonging to the same group indicated by the same colour.

The results of the PCA of the rhizosphere soil microbial communities (Figure 2A) revealed that, except for one SS point, all other points were clustered according to their respective soil types. The PCA of the root system endophyte communities (Figure 2B) showed that, except for two GSS points, all other points were clustered together according to their respective groups. After excluding these three outliers (one SS point and two GSS points), significant intergroup differences existed in the rhizosphere soil microbial communities and root system endophyte communities. Simultaneously, high similarity was maintained within groups. As shown in Table 4, the composition of the rhizosphere soil microbial community and root system endophyte community differed significantly among the three soil types (*p* < 0.05) (Ren et al., 2022a).

**Table 4:** Significance testing of rhizosphere soil microbial and root system endophyte community structures.

Category	Index	ADONIS		ANOSIM		MRPP	
		*F*	*p*	*R*	*p*	*δ*	*p*
Rhizosphere soil microorganisms	Jaccard	7.968	0.001	1.000	0.001	0.658	0.001
	Bray-Curtis	6.829	0.001	0.891	0.001	0.678	0.001
Root system endophytes	Jaccard	3.069	0.001	0.994	0.001	0.848	0.001
	Bray-Curtis	3.191	0.001	0.774	0.002	0.898	0.001

**Figure 3:**
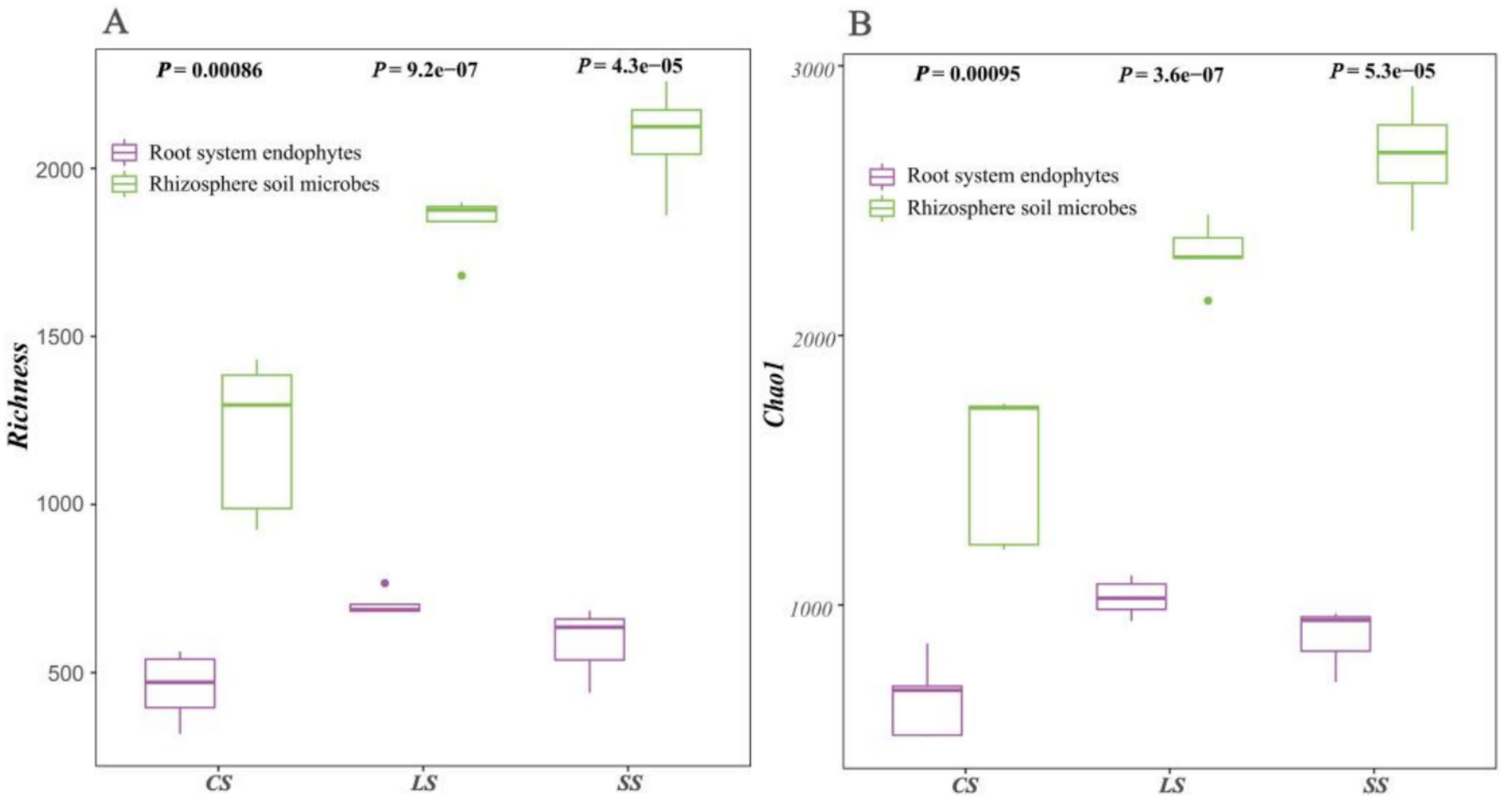
Alpha diversity analysis of rhizosphere soil microbes and root system endophytes. **(A)** Results for species richness. **(B)** Results for Chao1 estimated species richness index. Each point in the figure represented a biological sample, with samples belonging to the same group indicated by the same colour.

The results for the Alpha species diversity index of Richness (Figure 3A) and Chao1 (Figure 3B) of the microbial communities were similar, with both indicating that alpha diversity in the rhizosphere soil was consistently higher than that of the root system endophytes (*p* < 0.05). Alpha diversity of the rhizosphere soil microbes showed an increasing trend with changing soil type in the order of CS < LS < SS. Contrastingly, alpha diversity in the root system endophytes showed a slightly different pattern of change involving an initial increase and subsequent decrease, increasing in the order of CS < SS < LS.

**Figure 4:**
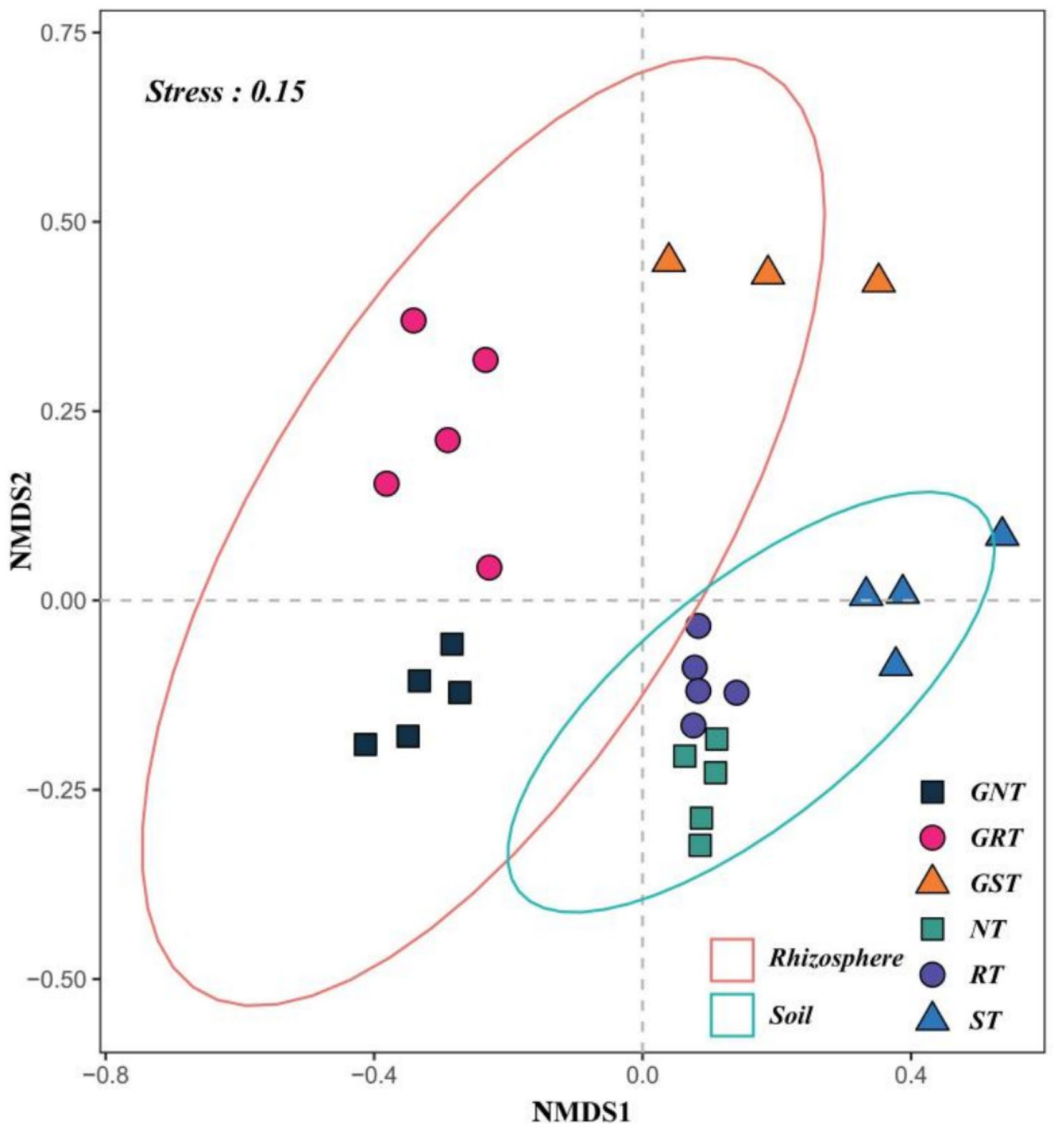
Beta diversity analysis of rhizosphere soil microbes and root system endophytes based on the non-metric multidimensional scaling (NMDS). Stress for rhizosphere soil microbes NMDS analysis was 0.056, and that for root system endophytes NMDS analysis was 0.091. Stress less than 0.1 shows good sorting; stress less than 0.05 shows that the analysis results are representative; stress less than 0.2 indicates that the NMDS analysis has certain reliability. The closer the sample on the coordinate diagram, the higher the similarity.

Figure 4 shows the analysis of beta diversity (Bray-Curtis distance) using the combined non-metric multidimensional scaling (NMDS) analysis of rhizosphere soil microbes and root system endophytes. The stress for rhizosphere soil microbes NMDS analysis was 0.056, and the stress for root system endophytes NMDS analysis was 0.091. A significant difference was found between the overall beta diversity of the two microbial communities (rhizosphere soil microbes and root system endophytes) (*p* < 0.05). The two confidence intervals represented the two microbial communities, and the distance between different points was a measure of their similarity or dissimilarity, with closer distances indicating higher similarity. Across different soil types, dissimilarities in microbes were greater in the root system endophyte community than those in the rhizosphere soil microbial community. Both microbial communities possessed similar structures, with the microbes in CS and endophytes in GCS exhibiting a shorter inter-community distance and higher degree of clustering with microbes in LS and endophytes in GLS, respectively, and a longer inter-community distance and higher degree of separation from microbes in SS and endophytes in GSS, respectively. These results demonstrate the presence of greater similarity between CS and LS in both the rhizosphere soil microbes and root system endophytes, with these communities showing higher similarity. CS and LS exhibited greater dissimilarity with SS, manifested as more significant differences in the microbial communities.

**Figure 5:**
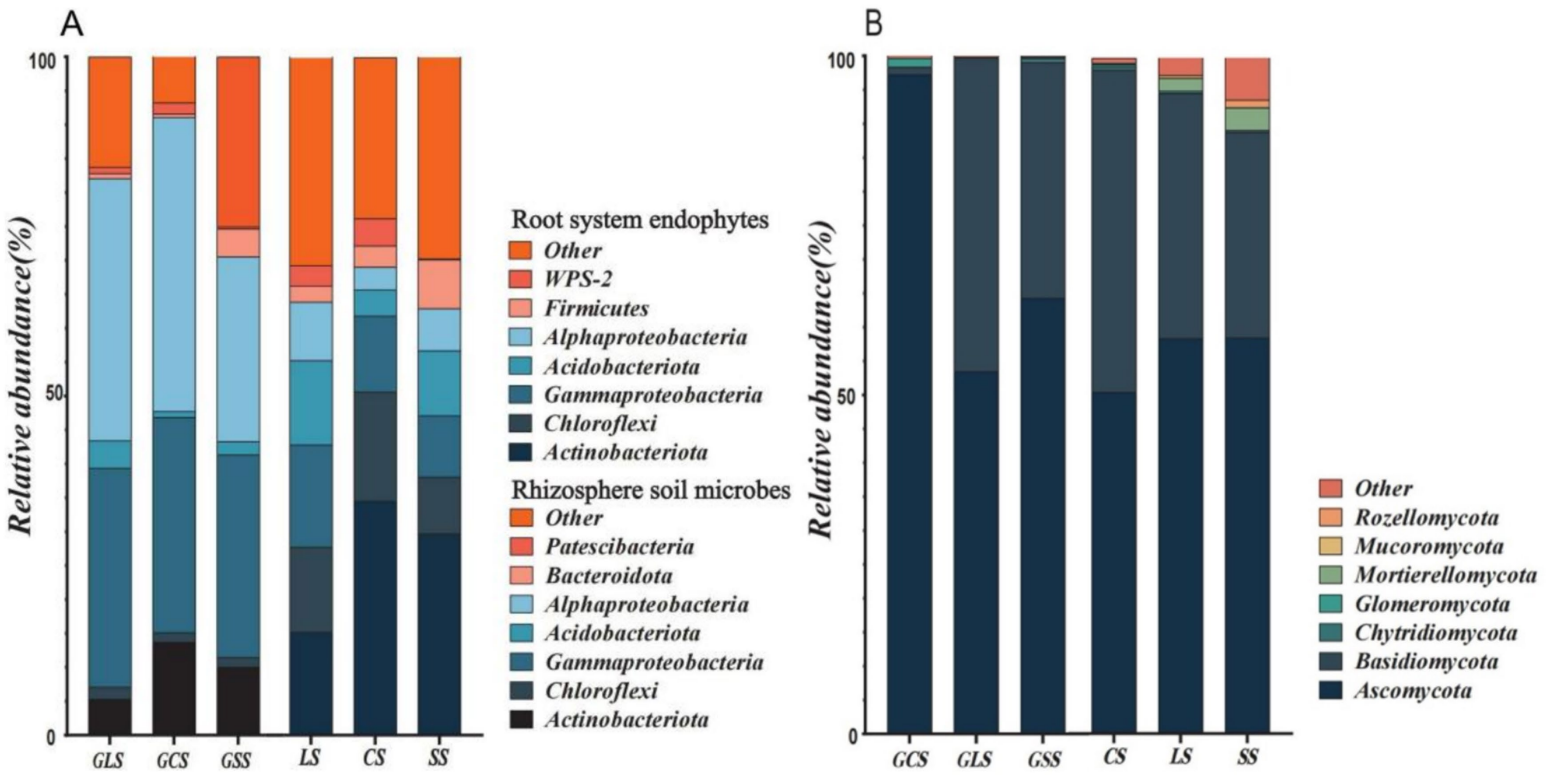
Relative abundance of root system endophytic and rhizosphere soil microbial species at the phylum level. **(A)** The relative abundance of bacteria. **(B)** The relative abundance of fungi.

Figures 5A,B show the relative abundance of bacteria and fungi at the phylum level in the root system endophytic species and rhizosphere soil microbial species. In the bacterial communities (Figure 5A), the top 5 dominant phyla for the root system endophytic and rhizosphere soil bacteria were identical and comprised *Actinobacteriota*, *Chloroflexi*, *Gammaproteobacteria, Acidobacteriota* and *Alphaproteobacteria*. However, the 6th and 7th most abundant phyla differed, with Firmicutes and WPS-2 for the root system endophytic bacteria and *Bacteroidota* and *Patescibacteria* for the rhizosphere soil bacteria; these results indicate that changes occurred in the microbial community composition from the rhizosphere soil to the root system endophytes, causing partial substitution among the dominant phyla.

The relative abundances of the top five dominant phyla also underwent changes. Proteobacteria (*Gammaproteobacteri*a and *Alphaproteobacteria*) were the dominant phyla in the root system endophyte community, accounting for >57% of bacteria in the entire microbial community and exhibiting a highly significant dominance. During the outward transition to the rhizosphere soil microbial community, considerable changes occurred in the pattern of dominance by Proteobacteria. Specifically, the relative abundance of Proteobacteria decreased, and significant changes occurred in the relative abundances of *Actinobacteriot*a and *Chloroflexi*. In this respect, the transition from the rhizosphere soil to the root system endophytes involved changes in the bacterial community composition and structure. The relative abundance of Proteobacteria in the top seven dominant phyla increased, whereas that of the other dominant phyla decreased; indicating that Gammaproteobacteria. *Alphaproteobacteria*, *Firmicutes* and WPS-2 could effectively colonise and grow the root system.

The diversity of bacterial species was also reduced during the transition from rhizosphere soil to root system endophytes, with the rhizosphere soil and root system endophytes containing 39 and 34 bacterial species (at the phylum level), respectively. *Actinobacteriota* in both the rhizosphere soil and root system endophytic bacterial communities exhibited a trend of increase in relative abundance in the order of LS < SS < CS (*p* < 0.05), this demonstrated that *Actinobacteriota* possessed different levels of adaptability to different soil types, and adaptability was the highest and lowest in CS and LS, respectively.

Within the fungal communities (Figure 5B), Ascomycota and Basidiomycota were the dominant phyla in the root system endophytic and rhizosphere soil fungi. Other phyla in the top seven dominant fungal phyla were *Chytridiomycota, Glomeromycota, Mortierellomycota, Mucoromycota* and *Rozellomycota*. Both fungal communities contained 13 fungal phyla (excluding unclassified phyla). In CS, the relative abundance of Ascomycota in the root system endophytic fungi was >97%, which was far higher than that in fungal communities under other soil conditions. However, the relative abundance of *Ascomycota* in the rhizosphere soil fungal community in CS was only >50%, indicating a considerable decrease in abundance. In CS and SS, the relative abundance of Ascomycota exhibited a decrease from the root system endophytes to rhizosphere soil, suggesting that Ascomycota were better adapted to growth in the root system endophytic environment than that in the rhizosphere soil. Among the three soil types, CS showed the best growth of Ascomycota, followed by SS and LS. *Mortierellomycota* also demonstrated different levels of growth adaptability in the three soil types, with growth adaptability increasing in the order of CS < LS < SS.

#### 3.3.2 Analysis of microbial community functions

**Figure 6:**
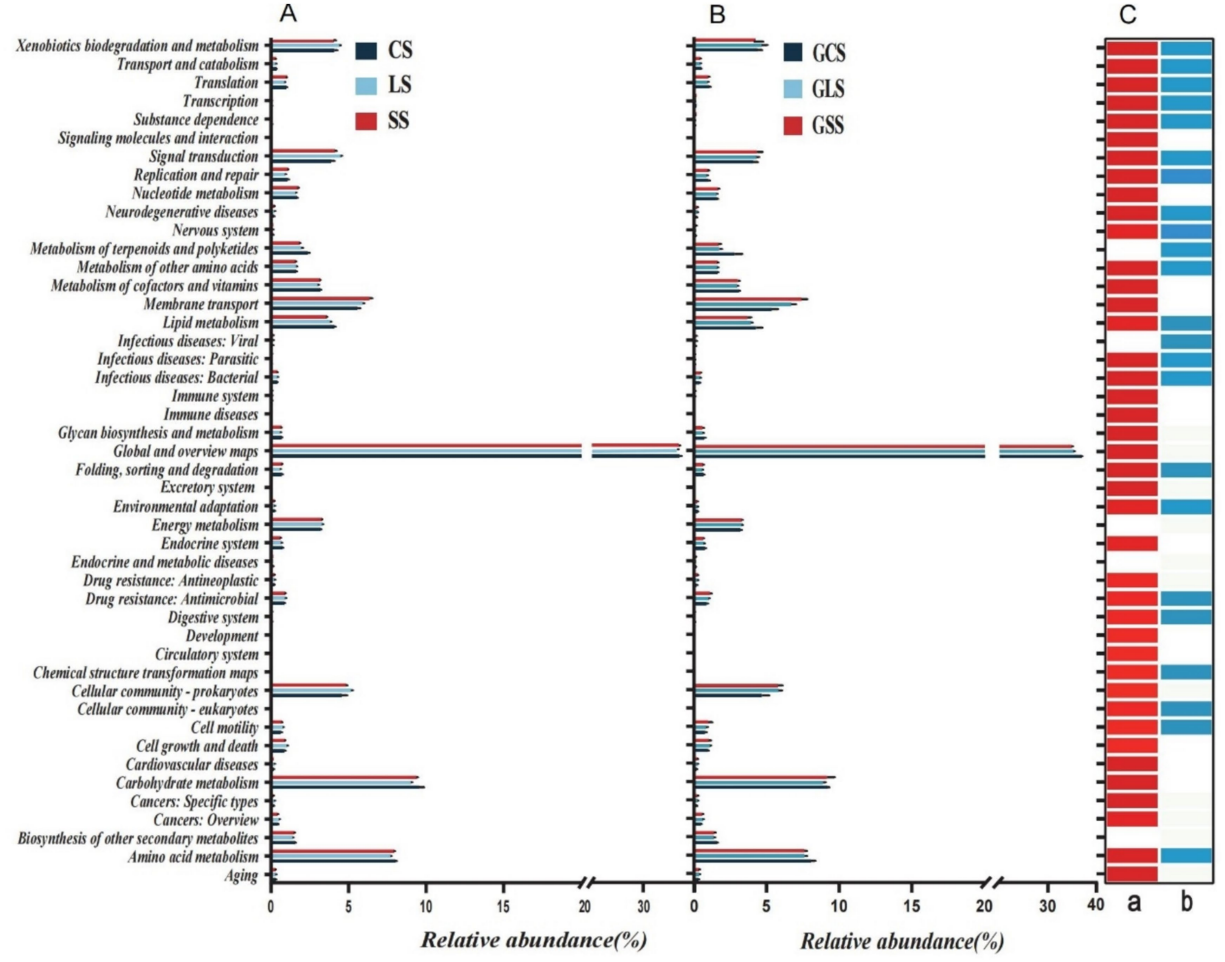
Relative abundances of 46 KEGG secondary metabolic pathways predicted by Tax4Fun2 in **(A)** rhizosphere soil microbes and **(B)** root system endophytes. **(C)** Results of the Kruskal-Wallis test for significant differences in mean values among different soil types for the two microbial communities, where (a) indicates the *p* values for functional differences among rhizosphere soil groups and (b) indicates the differences among root system endophyte groups. Red and light blue denotes *p* < 0.05, and white denotes *p* > 0.05.

Functions of all microbial communities were predicted using Tax4Fun2, with 46 KEGG secondary metabolic pathways obtained. The overall relative abundance distribution patterns of these pathways were similar between the rhizosphere soil microbes and root system endophytes. In each soil type, global and overview maps had the highest relative abundance (>34%), followed by carbohydrate metabolism (>8.8%), amino acid metabolism (>7.6%), membrane transport (>5.3%), cellular community-prokaryotes (>4.5%), xenobiotic biodegradation and metabolism (>3.7%) and lipid metabolism (>3.5%). These pathways showed high relative abundances in both types of microbial communities. Rhizosphere soil microbial community functions differed significantly across different soil types (*p* < 0.05) (Figure 6C: a). Among the 46 metabolic pathways, there was no significant difference among only 5 pathways in the rhizosphere soil for the different soil types, whereas there was no significant difference among 23 pathways in the root system endophytes for the different soil types, (Figure 6C: b) (*p* > 0.05). The biosynthesis of other secondary metabolites, endocrine and metabolic diseases and energy metabolism functions did not differ significantly in both microbial communities, indicating that soil type did not influence their metabolic capabilities. Across the different soil types, no significant differences in infectious diseases were observed (i.e., viral and the metabolism of terpenoids and polyketides) between the rhizosphere soil microbes. However, these differed significantly in the root system endophytes (*p* < 0.05), suggesting that soil type did not affect the expression of these two microbial community functions in the rhizosphere soil but it influenced the expression in the root system endophytes. For instance, the viral metabolic pathway is an antiviral toxicity pathway, which may be due to the interaction between rhizosphere soil microorganisms and endophytic bacteria in the root system, affecting the antiviral toxicity of different soil microorganisms (Figures 6A,B).

**Figure 7:**
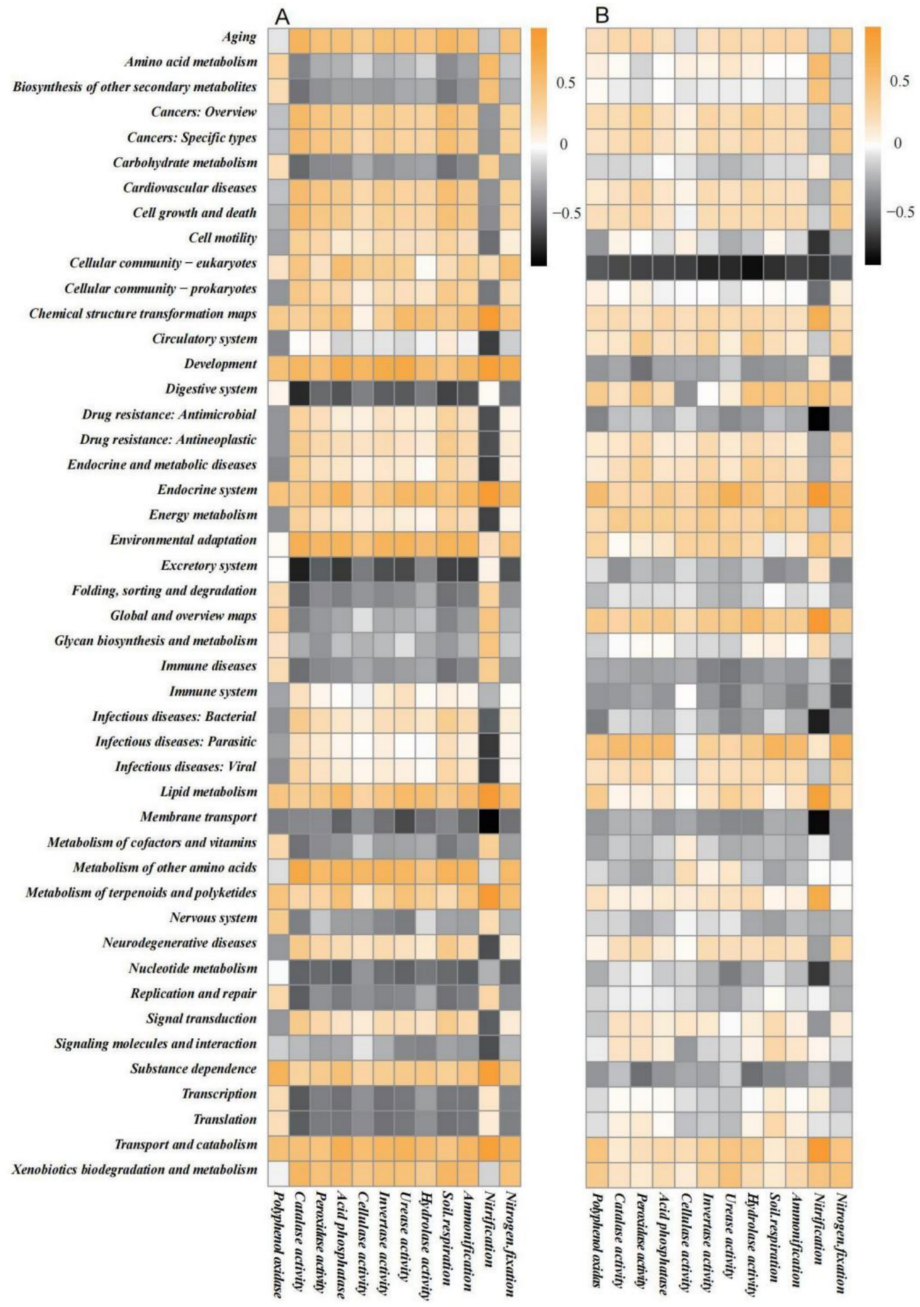
Spearman correlation heatmap of KEGG secondary metabolic pathways predicted by Tax4Fun2 in **(A)** rhizosphere soil microbes and **(B)** root system endophytes with soil biochemical functions.

Generally, correlations between rhizosphere soil microbes and soil biochemical functions (Figure 7A) were stronger than those between root system endophytes and soil biochemical functions (Figure 7B), suggesting that the metabolic activities of the former exerted a greater influence on soil biochemical functions. An analysis of the correlations between rhizosphere soil microbes and soil biochemical functions revealed that the amino acid metabolism, biosynthesis of other secondary metabolites, carbohydrate metabolism, digestive system, excretory system, folding, sorting and degradation, global and overview maps, glycan biosynthesis and metabolism, immune diseases, membrane transport, transcription, translation, nucleotide metabolism, replication and repair and metabolism of cofactors and vitamins pathways were all negatively correlated with most soil biochemical functions. The remaining metabolic pathways were mostly positively correlated or not correlated to soil biochemical functions. Nitrification showed a higher degree of correlation (positive or negative) with metabolic pathways such as transport and catabolism, substance dependence, membrane transport, and signalling molecules and interaction; this shows that nitrification was jointly regulated by multiple metabolic pathways, leading to complex correlations. Similar results were observed in other soil biochemical functions, such as nitrogen fixation.

#### 3.3.3 Correlations between microbial communities and environmental factors and sugarcane growth characteristics

**Figure 8:**
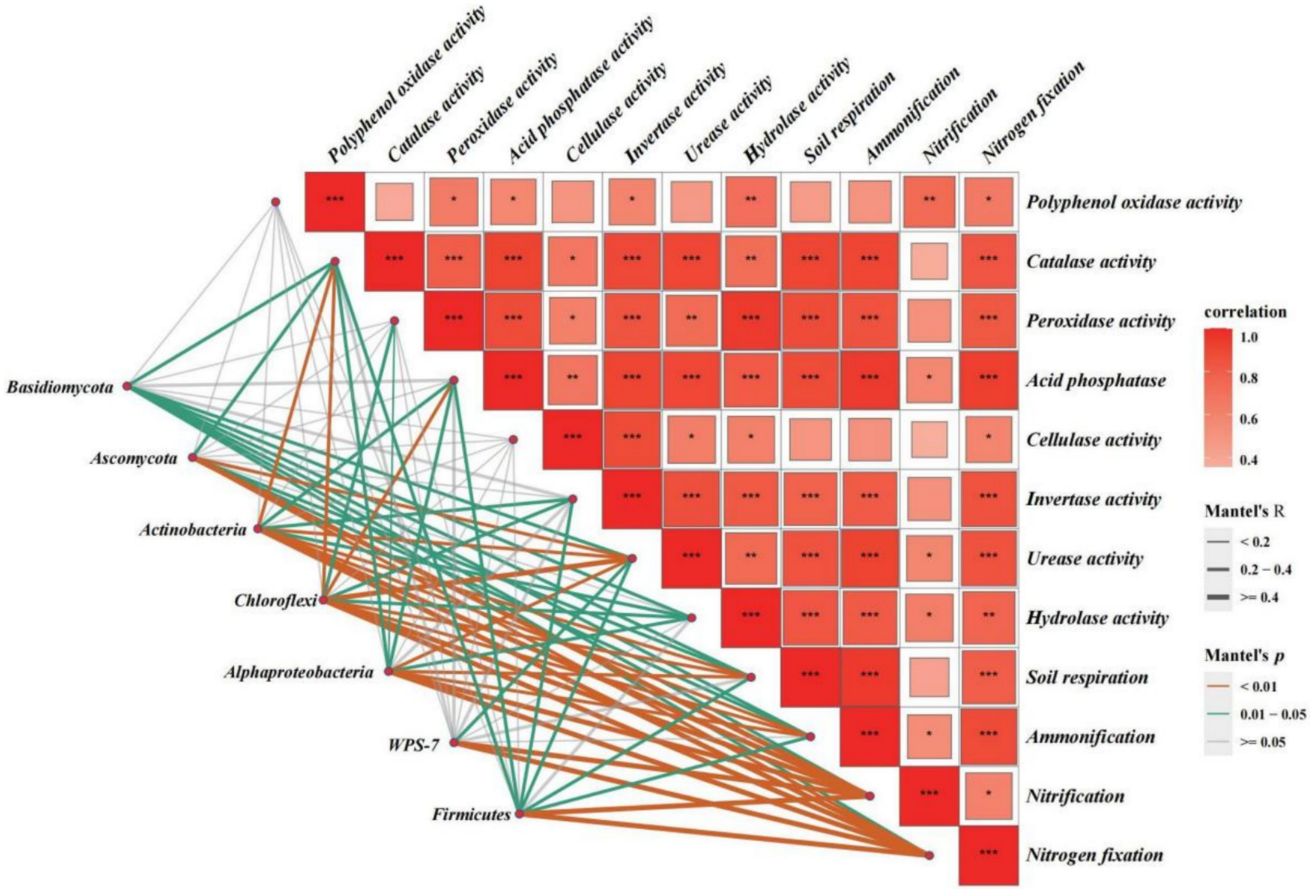
Results of the Mantel test were used to assess correlations between rhizosphere soil microbial species, the top seven relative abundances at the phylum level, and soil biochemical functions. The orange, blue, and grey lines denote Mantel *p* < 0.01, Mantel *p* < 0.05, and Mantel *p* > 0.05, respectively, and the thickness of the lines represents the correlation coefficient (R-value). Square boxes represent the correlations between soil biochemical functions, with red denoting a positive correlation and darker colours indicating a higher degree of correlation; *, **, and *** indicate *p* < 0.05, *p* < 0.01, and *p* < 0.001, respectively, for correlations between soil biochemical functions.

Figure 8 shows the results of the Mantel test used to assess the correlations between rhizosphere soil microbial species, the top seven relative abundances at the phylum level, and soil biochemical functions. We found that nitrification and nitrogen fixation in the rhizosphere soil were highly and significantly positively correlated with rhizosphere soil microbial species and the top seven relative abundances at the phylum level (except for Basidiomycota) (*p* < 0.01). Contrastingly, CL and PPO activity in the rhizosphere soil were not significantly correlated with rhizosphere soil microbial species and the top seven relative abundances at the phylum level, suggesting that these enzymatic activities were not influenced by the soil microbial community or were influenced by a smaller extent. Other soil biochemical functions were roughly influenced by certain species belonging to the top seven phyla, where correlations were statistically significant (*p* < 0.05).

**Figure 9:**
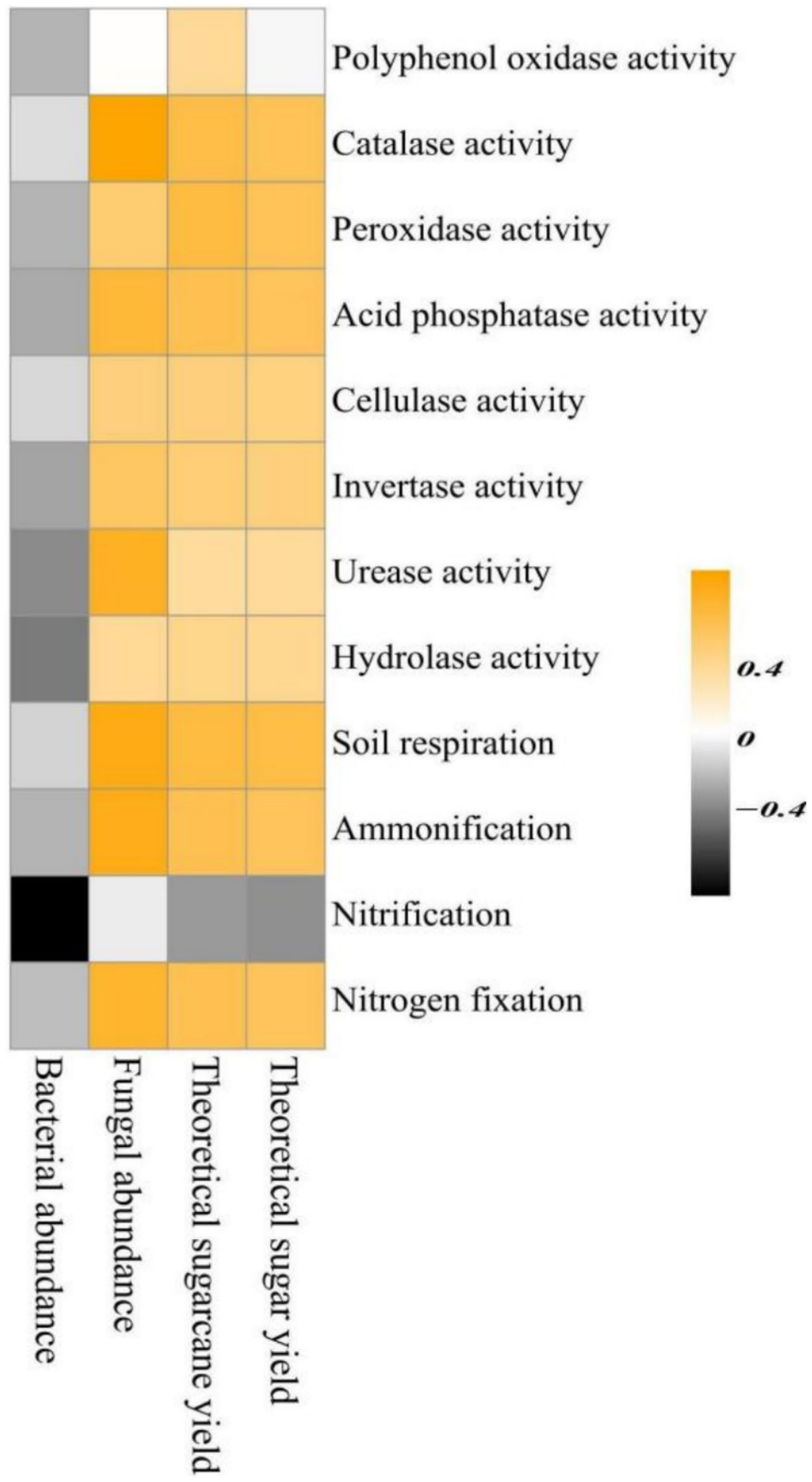
Spearman correlation analysis of rhizosphere soil microbial abundance and sugarcane yield indicators with soil biochemical functions.

The Spearman correlation analysis of rhizosphere soil bacterial and fungal abundance, theoretical sugarcane yield, and theoretical sugar yield with soil biochemical functions (Figure 9) indicated that rhizosphere soil bacterial and fungal abundance were negatively and positively correlated with 12 and 10 soil biochemical functions, respectively. However, fungal abundance was not correlated with nitrification and PPO activity (i.e., *R* = 0), indicating the presence of mutual promoting effects between fungal species abundance and soil biochemical functions. Considering the composition of rhizosphere soil fungal species shown in Figure 5, it can be observed that the top seven fungal phyla included many saprophytic taxa. Such fungi play a positive role in soil biochemical functions, are the main contributors to soil enzymatic activities, and can effectively promote the cycling of carbon, nitrogen, and phosphorus cycling. Positive correlations were found between the 2 sugarcane yield indicators (theoretical sugarcane and sugar yield) and 11 soil functions (soil enzymatic activities and biochemical functions, excluding nitrification); therefore, soil biochemical functions mainly influenced sugarcane yield by exerting promoting effects.

**Figure 10:**
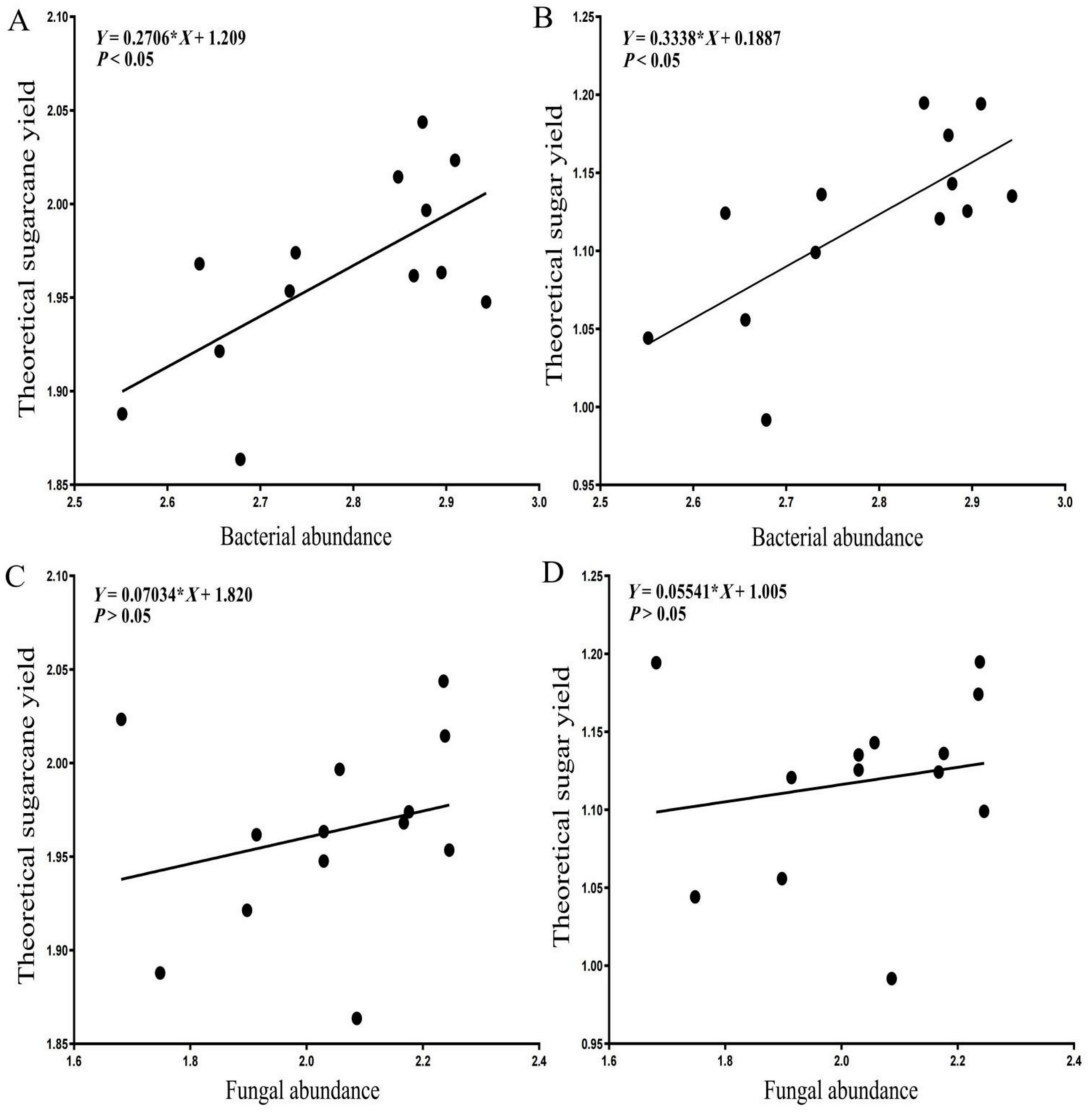
Linear regression analysis of root system endophytes with sugarcane yield indicators. **(A)** Linear regression between bacterial abundance and theoretical sugarcane yield. **(B)** Linear regression between bacterial abundance and theoretical sugar yield. **(C)** Linear regression between fungal abundance and theoretical sugarcane yield. **(D)** Linear regression between fungal abundance and theoretical sugar yield.

Linear regression analysis indicated that sugarcane root system endophytic bacterial abundance was significantly positively correlated with both the sugarcane yield indicators (*p* < 0.05), which increased with a rise in the alpha diversity of the root system endophytic bacteria (Figure 10). The root system endophytic fungal abundance was weakly positively correlated with the two sugarcane yield indicators, although the correlations were not statistically significant (*p >* 0.05), indicating that endophytic bacterial abundance in the root system of sugarcane was a significant factor influencing the theoretical sugarcane and sugar yields.

## 4 Discussion

### 4.1 Influence of non-microbial ecological factors in the sugarcane rhizosphere on sugarcane growth characteristics

Our study revealed positive correlations between soil biochemical functions and sugarcane growth characteristics. For instance, soil ammonification was highly significantly and positively correlated with POD, ACP and UE activities in soil, with correlation coefficients of 0.958, 0.927 and 0.911, respectively. For example, by regularly detecting enzyme activity in soil and using these indicators to evaluate soil health, it is possible to accurately guide the application of organic fertilizers, accelerate the ammonification process, and improve nutrient effectiveness. POD in the soil is a redox enzyme catalysing the hydrolysis of H_2_O_2_, thereby reducing the harm caused by H_2_O_2_ accumulation to the root systems of crops and promoting crop growth (Lu and Li, 2020). POD in the soil can catalyse the cycling of phenolic substances, such as aromatic compounds (Mei et al., 2021). Zhu et al. (2022) showed that a close correlation exists between soil enzyme activity and the number and composition of soil microorganisms, and soil enzyme activity and soil microbial community structure can be used as important references for soil fertility evaluation. Earlier research also reported that soil enzymes influence soil material cycling, optimizing the microbial community composition and diversity of rhizosphere soil, and increasing plant biomass and improving chemical quality (Reardon et al., 2022; Zhou et al., 2023). For example, by regularly detecting enzyme activity in soil and using these indicators to evaluate soil health, it is possible to accurately guide the application of organic fertilizers, accelerate the ammonification process, and improve nutrient effectiveness.

### 4.2 Influence of sugarcane rhizosphere microbial ecological factors on sugarcane growth characteristics

Reduced bacterial abundance could mean poor soil health, which could affects nutrient uptake and utilization by plants, which in turn affected their growth and yield (Bobul'ská et al., 2020). When the abundance of soil fungi is positively correlated with soil biochemical function, fungi play an important role in soil, such as participating in the decomposition of organic matter, the transformation and circulation of nutrients. An increase in fungal abundance may enhance these biochemical processes in the soil, favouring plant growth and development. Some fungi, such as arbuscular mycorrhizal fungi (AMF), can also form a symbiotic relationship with plant roots, which can promote the absorption of water and nutrients by the plant, thereby improving the plant’s stress resistance (Ge et al., 2022).

In this study, PCA and three methods were adopted to test and analyse differences in the microbial community structure composition between different groups. We found significant differences in the microbial community composition between the rhizosphere soil microbial and root system endophyte communities in the three different soil types. Simultaneously, high similarity was maintained within each group. Alpha diversity of the rhizosphere soil microbes was consistently higher than that of the root system endophytes. The alpha diversity of the rhizosphere soil microbes increased with soil type in the order of CS < LS < SS.

Contrastingly, the alpha diversity of the root system endophytes showed a slightly different pattern of change, increasing in the order CS < SS < LS. Therefore, greater homogeneity was present between CS and LS in both the rhizosphere soil microbes and root system endophytes, with both microbial communities showing higher similarity and both CS and LS exhibiting greater heterogeneity than those in SS. The top five dominant phyla of the root system endophytic and rhizosphere soil bacteria were identical. Ascomycota and Basidiomycota were the dominant phyla of the root system and rhizosphere fungi. These results suggest the presence of interactions and symbiotic relationships between species of the rhizosphere and endophyte communities of ratoon sugarcane. For example, some soil borne pathogens enter the root system from the rhizosphere soil, causing sugarcane disease.

The functions of all microbial communities were predicted using Tax4Fun2, and 46 KEGG secondary metabolic pathways were obtained. The overall relative abundance distribution patterns of these pathways were similar between the rhizosphere soil microbes and root system endophytes; this was similar to the those of findings reported by Cao et al. (2021), who suggested that rhizosphere soil microbes and root endophytes undergo co-evolution and growth and thus share a high similarity. Global and overview maps, carbohydrate metabolism, and amino acid metabolism were metabolic pathways with high relative abundances in both microbial communities. Rhizosphere soil microbial community functions differed significantly across different soil types. Among the 46 metabolic pathways, only 5 in the rhizosphere soil did not differ significantly between soil types, whereas half (23) of the pathways in root system endophytes did not differ significantly between soil types. Across different soil types, infectious diseases, such as viral and metabolism of terpenoids and polyketides, did not exhibit significant differences in the rhizosphere soil microbial community. Yet, they differed significantly in the root system endophytes, suggesting that soil type does not affect the expression of these two microbial community functions in the rhizosphere soil, but it influences the expression in the root system endophytes. Soil biochemical functions were more strongly correlated with rhizosphere soil microbes than those with root system endophytes, suggesting that the metabolic activities of the rhizosphere soil microbial community exert a greater influence on soil biochemical functions. A possible explanation for this is that plant root system endophytes indirectly regulate the rhizosphere microbial community by influencing the secretion of allelochemicals by the root system, which in turn affects soil biochemical functions (Wu et al., 2017). Some studies have proposed that “correlation does not equal causality” (Schoeler et al., 2016). Therefore, it is possible that some of our research findings have certain limitations and further experimental verification is warranted.

Nitrification and nitrogen fixation in the rhizosphere soil were significantly and positively correlated with the top seven relative abundances at the phylum level of rhizosphere soil microbial species (except for *Basidiomycota*). This correlation can be attributed to enhancements in soil physical and chemical properties, which in turn increase the relative abundance of dominant bacterial phyla in the soil, thereby promoting nitrification and nitrogen fixation activities. Contrastingly, CL and PPO activity in the rhizosphere soil were not significantly correlated with the top seven most abundant phyla, suggesting that these enzymatic activities were not influenced by the soil microbial community or were influenced to a lesser extent. Other soil biochemical functions were more or less influenced by particular species belonging to the top seven phyla, with statistically significant correlations. Rhizosphere soil bacterial abundance was negatively correlated with 12 soil biochemical functions, and fungal abundance was positively correlated with 10 soil biochemical functions. However, fungal abundance was not correlated with nitrification and PPO activity (i.e., *R* = 0), indicating the presence of mutual promoting effects among fungal species abundance and soil biochemical functions; this may be related to increased nitrogen and energy conversion in fungal pathways involved in the decomposition of organic matter in the sugarcane rhizosphere (Wang et al., 2021a). Ren et al. (2022b) reported that fungal abundance was positively correlated with the soil biochemical function-related soil *α*-glucosidase, N-acetyl-*β*-D glucosaminidase, and ACP activities, making it an environmental factor significantly influencing soil enzymatic activity, which agrees with the results of this study.

The fungal phyla with the top seven relative abundances included many saprophytic fungi. Such fungi play a positive role in soil biochemical functions; they are the main contributors to soil enzymatic activities and can effectively promote the cycling of carbon, nitrogen and phosphorus. Positive correlations were found between the 2 sugarcane yield indicators (theoretical sugarcane and sugar yields) and 11 soil functions (excluding nitrification): soil biochemical functions mainly influenced the sugarcane yield by exerting promoting effects. Bacterial abundance was significantly and positively correlated with the 2 sugarcane yield indicators, indicating that endophytic bacterial abundance in the root system of sugarcane significantly influenced theoretical sugarcane and sugar yields, and root system endophytic fungal abundance was weakly positively correlated with the two sugarcane yield indicators. This phenomenon may be related to several common fungal diseases in sugarcane, such as pineapple and red rot. These diseases primarily infect sugarcane seedlings, causing an inability to germinate and resulting in severe losses. Sugarcane smut, top rot disease and brown stripe disease are also fungal diseases infecting sugarcane buds, the bases of young leaves and tender shoot leaves (Li H. Y. et al., 2022; Li W. F. et al., 2022; Zhang et al., 2022). Overall, the findings of this study suggest that non-microbial and microbial conditions of rhizosphere created in different soil types significantly influence the growth characteristics of sugarcane. These results provide theoretical guidance for improving the soil composition and physical structure of sugarcane fields, as well as the application of chemical fertilizers, organic fertilizers, microbial agents and other amendments.

## 5 Conclusion

By integrating the results of field experiments, laboratory analyses, and high-throughput sequencing techniques, we conducted an in-depth study of the biochemical functions, enzymatic activities, and microbial ecological factors within the rhizosphere soil in sugarcane. This research provides comprehensive insights into the ecological characteristics of the rhizosphere in ratoon sugarcane across different soil types and their influence on the growth and yield of the crop. The ultimate goal of this integrated approach was to establish a scientific foundation for the sustainable cultivation of sugarcane. A positive correlation was observed between soil respiration, ammonification, nitrification, nitrogen fixation, soil enzyme activity and sugarcane growth characteristics. A positive correlation was also observed between various enzymes in the soil and the growth characteristics of sugarcane. We found symbiotic relationships between the rhizosphere soil microbial and root system endophyte communities, with significant functional differences evident between rhizosphere soil microbial communities from different soil types, confirming associations between functional expression and soil type. The abundance of bacteria and fungi in ratoon sugarcane rhizosphere soil was negatively and positively correlated with most soil biochemical functions, respectively, and correlations existed between the abundance of endophytic bacteria and fungi in the root system and sugarcane yield. Eleven soil biochemical and functional factors were positively correlated with theoretical sugarcane yield and theoretical sugar yield indicators. Our findings suggest that different feedback directions and correlation strengths exist between sugarcane growth characteristics and various ecological factors in their rhizosphere within different soil types across spatial scales.

## Data Availability

The original contributions presented in the study are publicly available. This data can be found here: https://www.ncbi.nlm.nih.gov/, accession number: PRJNA1247173. The raw data supporting the conclusions of this article will be made available by the authors without undue reservation.

## Footnotes

^1^ https://dataview.ncbi.nlm.nih.gov/object/PRJNA1247173?reviewer=83a59v9ec39p6lpsroc7tv8kum

^2^ https://dataview.ncbi.nlm.nih.gov/object/PRJNA1249922?reviewer=rbsrqu7r61n49bmr2lvm0ijlm0
