# Supplementary material for: Soil types create different rhizosphere ecosystems and profoundly affect the growth characteristics of ratoon sugarcane
Source: Front Microbiol. 2025 Jul 9;16:1541329. doi: 10.3389/fmicb.2025.1541329 (PMC12283674; doi:10.3389/fmicb.2025.1541329)
Supplement: Supplementary file 1 [file Data_Sheet_1.pdf]

Supplementary Table 1 Evaluation statistics of 338F_806R raw data

Sample	Valid reads	Average length	Total bases	Q30	Q20
GLS3	43027	413.64	17797669	94.36	98.26
CS2	64453	414.94	26743873	94.22	98.22
LS1	30105	412.41	12415548	94.52	98.32
GSS3	44476	411.20	18288542	94.38	98.25
GCS3	50436	410.48	20703045	94.66	98.34
GLS5	49206	411.56	20251080	94.77	98.38
GSS5	47472	417.19	19805017	94.64	98.37
GSS2	50708	413.34	20959691	94.60	98.31
LS3	55142	413.36	22793313	94.09	98.10
LS2	56035	412.30	23102960	94.53	98.32
GLS4	45494	411.49	18720201	94.76	98.39
CS5	59428	411.69	24465834	94.05	98.05
SS3	70978	416.72	29578001	94.22	98.22
GCS1	45659	413.87	18896790	94.60	98.34
LS4	37414	412.13	15419387	94.48	98.28
SS4	60905	415.66	25315773	93.90	98.02
GSS1	47868	411.72	19708065	94.47	98.22
LS5	51154	412.79	21115994	93.96	98.15
CS1	59602	412.00	24555838	93.93	98.12
CS3	54178	412.07	22324926	94.01	98.15
GCS2	49651	411.40	20426663	94.41	98.17
GSS4	41923	425.35	17832124	94.49	98.38
GCS5	48449	412.21	19971103	94.13	98.18
SS5	69914	415.88	29075634	94.36	98.24
GLS2	43305	414.22	17937711	94.16	98.21
SS1	62532	415.25	25966472	94.46	98.31
CS4	52328	415.02	21717427	94.49	98.33
GCS4	51606	411.08	21214086	94.31	98.26
SS2	69963	416.52	29140799	94.08	98.17
GLS1	42716	410.26	17524629	94.98	98.45

Supplementary Table 2 Evaluation statistics of ITS1F_ITS2R raw data

Sample	Valid reads	Average length	Total bases	Q30	Q20
GLS2	33031	240.96	7959259	99.04	99.63
CS2	72901	214.85	15662712	98.73	99.40
GLS5	49584	228.65	11337135	99.41	99.77
SS3	55282	247.40	13677014	98.28	99.27
GSS2	41820	236.91	9907725	99.11	99.67
SS5	64061	226.60	14516265	99.20	99.66
GLS3	56424	259.79	14658312	98.87	99.53
GLS4	44147	249.39	11009705	98.56	99.40
CS5	64878	219.29	14227342	98.90	99.53
GSS3	49777	252.75	12580970	98.77	99.42
GCS5	47985	240.36	11533664	98.91	99.52
LS3	63933	246.80	15778811	98.20	99.19
GSS4	53641	237.27	12727210	99.41	99.77
SS1	65546	243.52	15961659	98.86	99.52
LS1	70271	241.16	16946418	98.58	99.38
CS1	73999	208.66	15440341	99.00	99.56
GSS1	40395	266.28	10756245	99.15	99.70
CS4	71670	230.91	16549470	98.58	99.38
GSS5	57237	235.51	13479648	99.04	99.61
GCS3	64691	245.35	15871926	98.33	99.28
LS5	74203	215.50	15990462	98.96	99.52
SS4	59189	238.09	14092047	99.07	99.61
SS2	72978	223.67	16322814	99.36	99.74
GCS2	61199	255.16	15615233	98.02	99.14
LS2	68043	243.82	16590397	98.20	99.22
GCS1	61313	239.95	14712067	98.77	99.49
LS4	71885	238.18	17121713	98.54	99.34
GCS1	60304	252.52	15227980	97.99	99.11
CS3	72785	237.04	17252801	98.40	99.30
GLS1	61080	262.85	16055023	98.49	99.36
